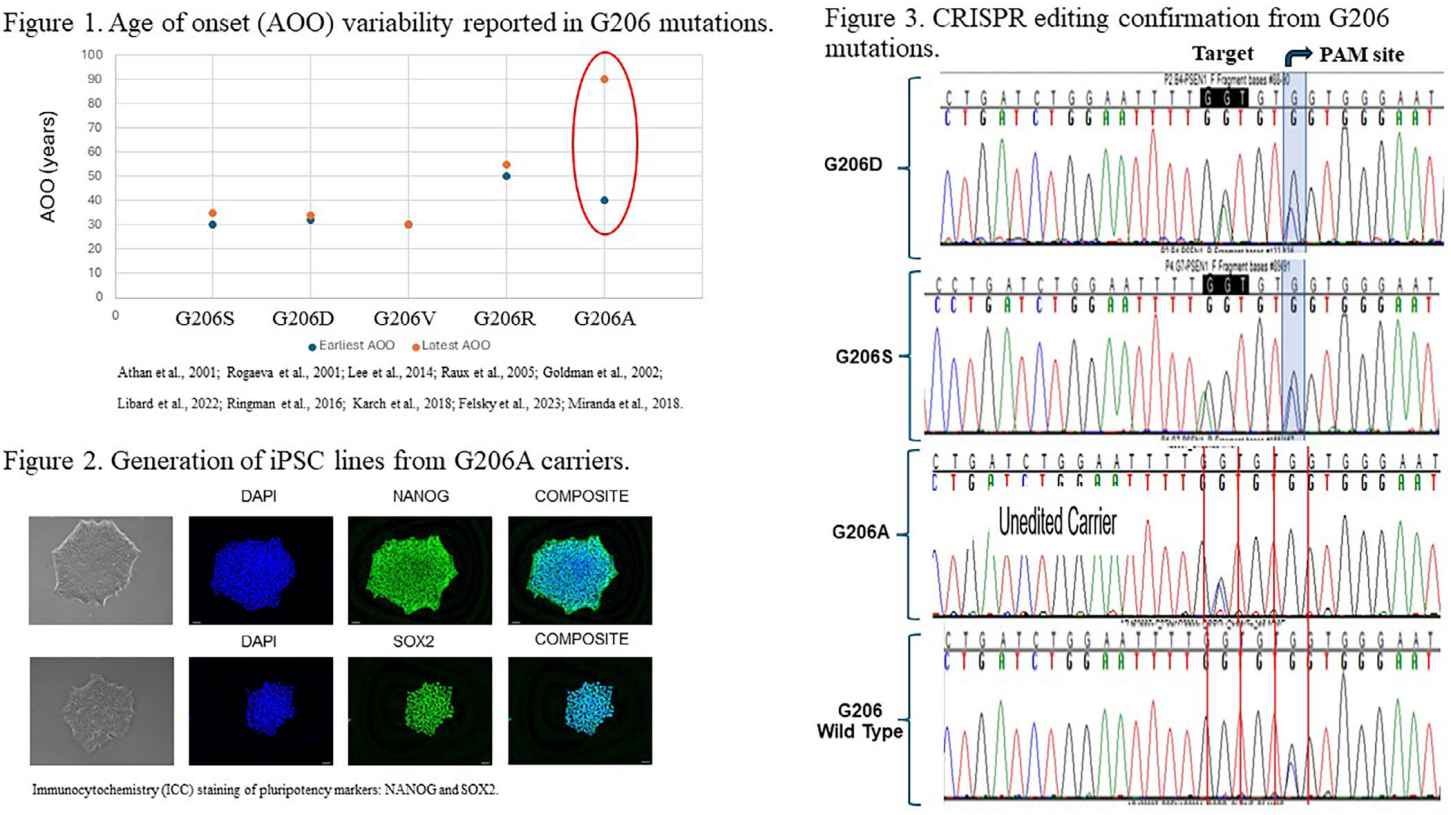# Molecular and functional analysis using Presenilin‐1 G206A mutation iPSC lines to understand age‐of‐onset variability in autosomal dominant Alzheimer disease

**DOI:** 10.1002/alz.091393

**Published:** 2025-01-03

**Authors:** Katrina Celis

**Affiliations:** ^1^ John P. Hussman Institute for Human Genomics, Miller School of Medicine, Miami, FL USA

## Abstract

**Background:**

Pathogenic variants in Presenilin‐1 (*PSEN1*) cause autosomal dominant forms of early‐onset Alzheimer disease (AD). Despite the high penetrance, substantial interindividual variability in age‐of‐onset (AOO) is observed among different *PSEN1* mutations. Attempting to understand the molecular mechanisms leading to variability in AOO in *PSEN1* mutations, we focused on three mutations located at codon 206 (G206A, G206S and G206D) of this gene. The G206A variant is associated with a wide range AOO, 30‐90 years, while G206S and G206D have a less variable AOO reported in only a few cases (30‐35 years). Molecular and functional analysis from induced pluripotent stem cells (iPSC) carrying these mutations may help us identify the transcriptomic changes and cellular pathways associated with AAO variability.

**Method:**

We identified 46 carriers of the G206A mutation using whole genome sequencing from 182 families recruited as part of the Puerto Rican Alzheimer Disease Initiative (PRADI). We collected peripheral blood mononuclear cells (PBMCs) for iPSC generation from 19 of those 46 PRADI G206A carriers. We selected six G206A carriers, three with AOO <60 and three with AOO >65 years. CRISPR/Cas9 genome editing technologies were used to introduce the G206S, G206D and G206WT (wild type) variants from a G206A iPSC line. All eight lines (six G206A, one G206S and one G206D) will go under neuronal differentiation and spheroid generation protocol for transcriptomic, proteomic and AD‐related functional analysis.

**Result:**

We have successfully reprogrammed six iPSC lines carrying the G206A mutation. CRISPR genome editing has been performed for the G206D and G206S variants. and confirmed by Sanger sequence. Next steps include neuronal differentiation and spheroids generation from iPSC lines, followed by bulk RNA sequencing from neurons, single cell RNA sequencing from spheroids and AD functional analysis including assays for Aβ40, Aβ42, APP, Tau, and NOTCH.

**Conclusion:**

Our iPSC lines will be instrumental to identify potential molecular determinants of AOO in *PSEN1* mutation carriers. Transcriptomic and functional data is in process and will be presented at the conference. These data will be informative for the identification of molecular changes associated with *PSEN1* mutations in neurons and other cell types that will further increase our understanding of *PSEN1* role in AD pathogenesis.